# Impact of Fertilisation on the Bacterial Core Microbiome of Grassland Soils: Abundance in the Field and Growth In Vitro

**DOI:** 10.1111/1758-2229.70235

**Published:** 2025-11-21

**Authors:** Rostand R. Chamedjeu, Kunal Jani, Karoline Jetter, Kerstin Wilhelm, Patrick Schäfer, Lena Wilfert, Simone Sommer, Christian U. Riedel

**Affiliations:** ^1^ Microbial Biotechnology, Department of Biology University of Ulm Ulm Germany; ^2^ Institute of Evolutionary Ecology and Conservation Genomics University of Ulm Ulm Germany; ^3^ Institute of Phytopathology, Research Centre for BioSystems, Land Use and Nutrition Justus Liebig University Giessen Germany

**Keywords:** 16S rRNA gene amplicon sequencing, bacterial diversity and biomarkers, grassland ecosystems, organic fertilisation, soil microbiome

## Abstract

Anthropogenic activities may have profound impacts on the soil microbiome with consequences for soil health, agriculture and food production. Here, we investigated the impact of different fertilisation regimes on the composition of the bacterial soil microbiome in grassland ecosystems by 16S rRNA gene amplicon sequencing and in vitro growth experiments with culturable representatives of the bacterial core microbiota. We observed a large proportion of taxa shared across fertilisation regimes without significant differences in their evenness, but shifts in the composition of the bacterial core microbiome by fertilisation. These effects were most pronounced for fertilisation with pig slurry (PS). Analysis of microbiome multivariable association with linear models identified bacterial biomarker taxa for different fertilisation regimes. This enabled the selection of several culturable representatives for in vitro growth experiments. Consistent with the relative abundances of *Bradyrhizobium*, *Nocardioides*, and *Solirubrobacter* in field samples, the growth of *Bradyrhizobium japonicum* was inhibited by PS, while *Nocardioides albus* and *Solirubrobacter pauli* exhibited enhanced growth in its presence. Our results suggest that culturable representatives of the bacterial core soil microbiota can be identified and used to investigate the effects of specific parameters linked to anthropogenic impacts under controlled laboratory conditions.

## Graphical abstract

Effects of different organic fertilisation regimes on the bacterial microbiome of grassland soil were analysed by 16S rRNA amplicon sequencing. Different fertilisation regimes led to distinct changes in the composition of the bacterial microbiome and effects were most pronounced when soil was fertilised with pig slurry. In in vitro growth experiments, the response of culturable representatives of the bacterial core microbiome to pig slurry mirrored their changes in abundances in the field.

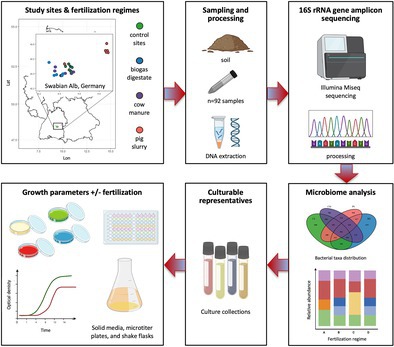

## 1 Introduction

Soil provides numerous ecosystem functions and services, including plant growth, nutrient cycling, carbon sequestration, and water purification (Timmis and Ramos 2021; Kopittke et al. 2024). As such, soil health is crucial for sustainable agriculture, reducing the risk of soil degradation and supporting resilience against environmental changes (Kopittke et al. 2024; Raupp et al. 2024). The microbiome is a major biological driver of ecosystem functions and services of soil, and its activity is influenced by abiotic factors, such as soil physico‐chemical properties, as well as external factors like agricultural land use and fertilisation (Bissett et al. 2011; Philippot et al. 2024). In addition, soil microorganisms play a central role in terrestrial biogeochemistry, driving the cycling of soil organic matter, which represents the largest primary source of plant nutrients (Fierer 2017; Yadav et al. 2021). Previous studies have demonstrated a positive correlation between the diversity of the soil microbiome and a range of ecosystem functions including nutrient cycling, decomposition of soil organic matter, plant development, growth and protection against pathogens (Delgado‐Baquerizo et al. 2020; Trivedi et al. 2022). In addition, the soil microbiome also represents an important genetic resource for global health (Lehmann et al. 2020; Wood and Blankinship 2022). For example, many soil microbes harbour biosynthetic gene clusters related to the production of antimicrobials, and their diversity is of relevance to address the current crisis of antimicrobial resistance (de Castro et al. 2014; Ma et al. 2023; Zhang et al. 2024).

Gradual and persistent anthropogenic impacts can cause shifts in the diversity and activity patterns of the soil microbiome and thus may have consequences for the functional stability of the soil ecosystem (Siebert et al. 2019; Thakur et al. 2020). Land‐use intensification represents one of the major anthropogenic factors that alter soil biodiversity and affect ecosystem processes (Matson et al. 1997; Babin et al. 2021). In agricultural ecosystems under intensified human land use, loss in biodiversity and decline in ecosystem functioning are associated with significant soil degradation, loss of productivity and increased greenhouse gas emissions (Hurni et al. 2015). For example, conventional tillage is deleterious for soil physicochemical and biological properties (Congreves et al. 2017), which may have consequences in soil microbiome structures. It is also known that different management practices in agroecosystems are associated with different diversity patterns of the soil microbiome. However, the effects of different organic fertilisation regimes on microbial diversity in soil and the consequences on ecosystem functions are currently poorly understood impeding our ability to develop adapted management systems for sustainable agricultural land use.

A deeper understanding of soil microbial communities, often referred to as the *microbiome* or *microbiota*, is essential to comprehend their contribution to these vital ecosystem functions. While the terms *microbiome* and *microbiota* have partially overlapping definitions and are often used interchangeably, they have distinct definitions (Berg et al. 2020). The *microbiome* is typically referred to as “a characteristic microbial community occupying a reasonably well‐defined habitat with distinct physio‐chemical properties,” encompassing not only the microorganisms but also their metabolic activities. By contrast, *microbiota* refers specifically to the assembly of microorganisms across different kingdoms, including Prokaryotes (Bacteria, Archaea) and Eukaryotes (e.g., Protozoa, Fungi, and Algae). This distinction is important for understanding the complex dynamics within soil ecosystems and how they may be influenced, for example by agricultural practices such as fertilisation.

One approach to evaluate the impact of various factors on the condition and functions of an ecosystem is to analyse biomarker organisms or communities and their responses (Gerhardt 2002). More recently, ‘next‐generation biomonitoring’ has been used to analyse changes in diverse ecosystems including soil by sequencing entire genetic information using next‐generation technologies and machine learning algorithms (Bohan et al. 2017; Fierer 2017; Cordier et al. 2019, 2021). In this study, we investigate the impact of different organic fertilisation regimes on the composition of the bacterial soil microbiome in grassland ecosystems. We hypothesized that different organic fertilisation regimes have distinct effects on the composition and functional diversity of the bacterial grassland soil microbiome by enriching and/or depleting specific taxa. Focusing on the bacterial components of the soil microbiome, we sequenced 16S rRNA gene amplicons to identify representative species that may then be used as biomarkers for fertilisation status. Additionally, we sought to test whether the effects of fertilisation can be replicated in in vitro growth experiments with representative, culturable type species of these biomarkers. This approach may help to better understand the impact of organic fertilisation on soil microbial dynamics and improve predictions of the responses of agricultural ecosystems to anthropogenic disturbances. Furthermore, this may support the development of more robust biomonitoring methods and promote sustainable management of ecosystem services in grassland ecosystems affected by intensive land use or other environmental stresses.

## 2 Materials and Methods

### 2.1 Study Sites and Sampling Design

**FIGURE 1:**
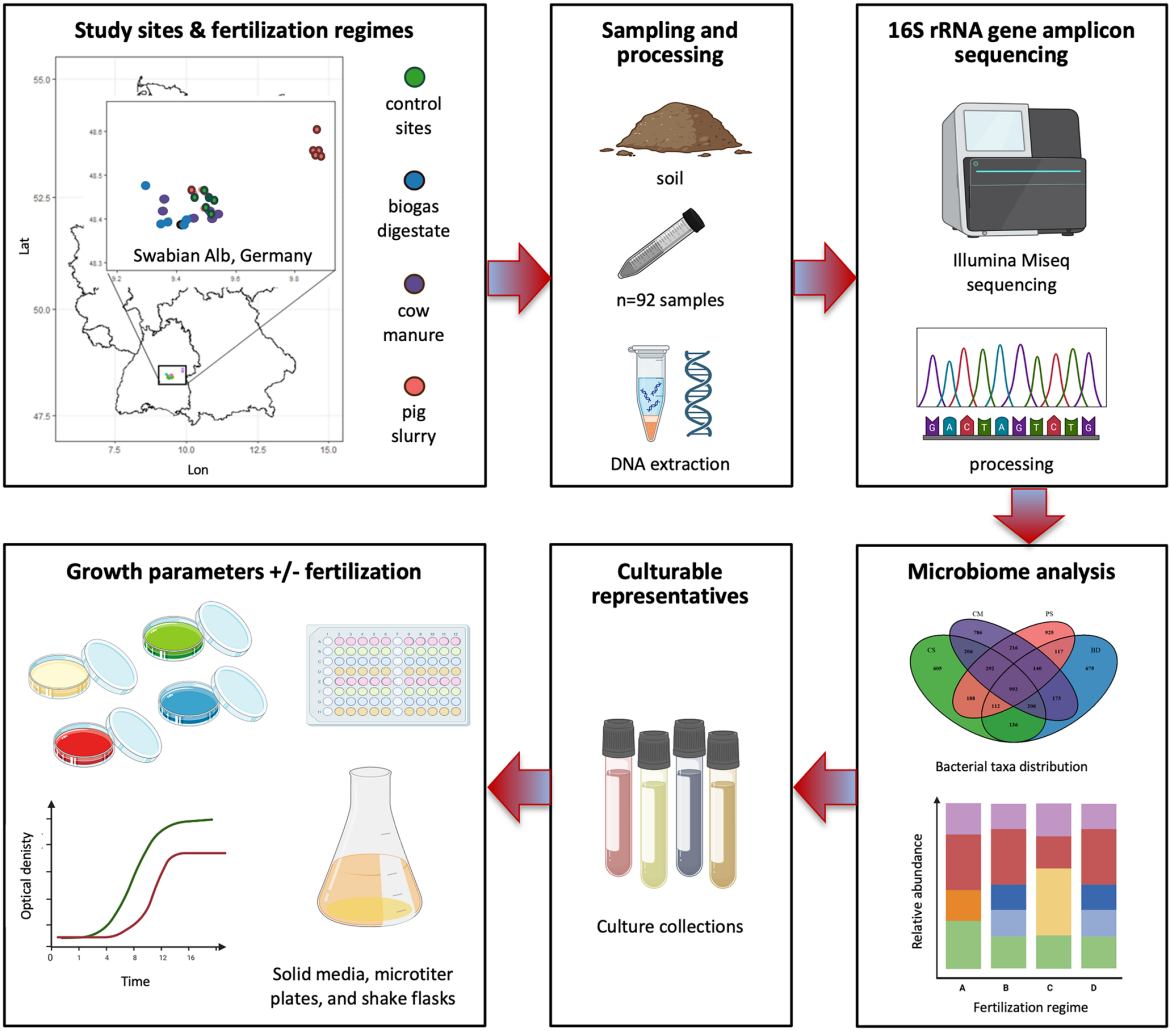
Workflow of the presented study with a map of the study sites for field sample collection, experimental setup, sample processing, 16S rRNA gene amplicon sequencing, analysis of the bacterial microbiome, and selection of culturable representatives of the bacterial core microbiome to test the effects of fertilisation on growth in vitro.

Soil samples were collected from grassland ecosystems on the Swabian Alb, Germany (Figure 1: Graphical abstract). The soils in this region are primarily loamy and silty clays with high skeletal content. The farming systems in the study area follow specific regimes of mowing, grazing, and organic fertilisation. The experimental design included study sites managed for several years by four different fertilisation regimes (*n* = 5–6 study sites per management regime). Three groups of study sites were intensively managed with several rounds of fertilisation and mowing per year. The study sites were either fertilised with pig slurry (PS), cow manure (CM), or biogas digestate (BD). Control soil (CS) was obtained from a fourth group of sites mowed only once a year and fertilised exclusively by occasional grazing by flocks of sheep (1–2× per year). Except for PS sites, all sites are part of the Biodiversity Exploratories “Schwäbische Alb” (https://www.biodiversity‐exploratories.de/en/). Soil samples were collected between May and July 2022 from the top‐soil at 0–20 cm depth. Per site, four samples were collected from randomly selected spots. Before further processing, stones and plant debris were removed by passing samples through a sieve (mesh size 0.1 cm). Samples were immediately placed in a cooling box, transported to the laboratory, and stored at −80°C until further processing. In total, 92 samples (4 regimes, 5–6 sites per regime, 4 biological replicates per site) were obtained for 16S rDNA gene amplicon sequencing.

### 2.2 DNA Extraction and Sequencing

Total DNA was extracted from ~200 mg of soil sample using the ZymoBIOMICS DNA Miniprep Kit (Zymo Research, USA) following the manufacturer's instructions. DNA concentration was determined after extraction and ~5–20 ng of DNA was used for amplification of the V4 region of 16S rRNA genes as described elsewhere (Fackelmann et al. 2021). In brief, a 291 bp fragment was amplified using the 515F (′5‐GTGCCAGCMGCCGCGGTAA‐3′) and 806R primers (5′‐GGACTACHVGGGTWTCTAAT‐3′) (Caporaso et al. 2011) following the earth microbiome protocol and recommendations for polymerase chain reaction (PCR) amplification. This first PCR included 1 μL template DNA, 5 μL AmpliTaq Gold 360 Master Mix (Applied Biosystems, Darmstadt, Germany), 1.5 μL TS primers, and 2.5 μL water to give a final volume of 10 μL. The amplification protocol included an initial denaturation of 10 min at 95°C, followed by 30 cycles with 95°C for 30 s, 58°C for 30 s and 72°C for 45 s, followed by a final elongation of 72°C for 7 min. In a second PCR, amplicons were barcoded. Primers for barcoding were tagged with universal adapters (CS1 and CS2, Standard BioTools, South San Francisco, USA) and 4 Ns were added to the forward primer for cluster identification during sequencing. PCR reactions contained 3 μL template amplicon from the unbarcoded sample, 4 μL individual barcodes and Illumina adapters (Fluidigm Access Array System for Illumina Sequencing Systems, Standard BioTools, South San Francisco, USA), and 3.0 μL ultrapure water for a total of the 20 μL reaction. The reaction times were identical to the first PCR, but only 10 cycles were carried out. All PCRs were performed on a SimpliAmp Thermal Cycler (Applied Biosystems, Darmstadt, Germany) with negative controls containing only the reagents included and subsequently sequenced.

Barcoded PCR products were purified to remove residual oligonucleotides using the NucleoMag NGS Clean‐up and Size Select Kit (Macherey‐Nagel, Düren, Germany) on a GeneTheatre (Analytik Jena, Jena, Germany) according to the manufacturer's guidelines and in‐house workflow and checked for expected amplicon length using capillary electrophoresis on a QIAxcel Advanced System (QIAGEN, Hilden, Germany). DNA concentrations of the barcoded samples were quantified using the QuantiFluor dsDNA System (Promega, Madison, USA) on a TECAN Infinite F200 PRO plate reader (Tecan, Männedorf, Switzerland). Samples were normalised to include 60 ng of each indexed amplicon in the final library. For sequencing, 8 pM of the libraries were loaded onto a MiSeq flow cell and spiked with PhiX sequencing control V3 at 5% (Illumina MiSeq Reagent Kit V2). Paired‐end sequencing was performed over 2 × 251 cycles in an Illumina MiSeq (Illumina, San Diego, USA) following the manufacturer's recommendations.

### 2.3 Analysis of the Bacterial Soil Microbiome

Raw reads were analysed in QIIME 2 v2022.8 (Bolyen et al. 2019). DADA2 (Callahan et al. 2016) was used for quality filtering and assembly of reads into amplicon sequence variants (ASVs) and SILVA v138.2 was used as a taxonomic reference database. ASVs, taxonomy, metadata and phylogeny were imported into R package phyloseq v1.40.0 (McMurdie and Holmes 2013). ASVs identified in the blanks and controls were removed from samples to avoid false results and ASVs classified as mitochondria, chloroplasts and archaea were filtered out. We applied an additional filter to remove rare ASVs with fewer than 10 reads across the entire dataset. The raw reads were submitted to the NCBI database (BioProject: PRJNA1188490). Samples analysed in this study are the subset consisting of the soil samples and associated accession numbers are provided in Table S1.

All downstream analyses and visualisation were performed in R v4.2.2 (R Core Team 2022). To reflect the inherent spatial variation of bacterial communities within the experimental sites as well as to avoid observations based on singularities, we analysed individual soil samples nested within sites. The impact of organic fertilisation on soil microbial communities was investigated by analysing the distribution of taxa among fertilisation regimes. Absolute numbers of taxa unique to or shared by different experimental groups were extracted using the ‘get_vennlist’ function and were plotted with the R package VennDiagram v1.7.3 (Chen and Boutros 2011). Pielou's evenness indices of shared taxa were calculated for each fertilisation regime using the ‘diversity’ function of the R package VEGAN v2.6.4 (Dixon 2003). The effect of fertilisation regime on species evenness was assessed by fitting a generalised linear mixed model (GLMM) with a Gamma distribution (log link). To account for the hierarchical sampling design, treatment was included as a fixed effect, while site and sample (nested within sites) were specified as random intercepts. Core taxa were selected among shared soil microbial communities by applying cut‐off values for prevalence (present in at least 50% of all samples) and abundance (detection threshold > 0.01% relative abundance) using the ‘subset_taxa’ function of the phyloseq R package and only genera with valid taxonomic descriptions were retained. Differences in the relative abundances of core bacterial members were assessed using GLMMs with abundances transformed for beta regression and modelled with a beta distribution (log link), with samples nested within site as a random factor. Heatmaps clustering ASVs according to prevalence across all samples and relative abundance within samples were generated using the ‘heatmap’ function of the R package ComplexHeatmap v2.9.2 (Gu et al. 2016). Additionally, to identify potential fertilisation‐responsive bacterial biomarkers, we used the MaAsLin2 package v1.12.0 (Mallick et al. 2021) with the same model structure as described above (samples nested within sites). Core bacterial taxa were agglomerated prior to analysis, and the data were normalised using total sum scaling and log‐transformation. PICRUSt2 v2.5.2 (Douglas et al. 2020) was used to predict the functional potential of the bacterial biomarkers at the study sites. This software package allows predictions on the functional potential of a bacterial community based on abundance profiles of marker gene sequences such as 16S rRNA gene amplicons. The obtained KEGG orthologs were used to compute functional diversity and its response to various fertilisation regimes. To test the effect of fertilisation‐induced changes in the bacterial community on key metabolic pathways, a GLMM using a Gamma distribution (log link) was applied, again with treatment as a main effect and sample nested within site as a random factor. Finally, correlation between the bacterial biomarkers and functional diversity was tested by Spearman correlation using the ‘cor_test()’ function in base R, which accounts for the difference in the data distribution for various biomarker bacteria. All R code used for statistical analysis and visualisation is publicly available on GitHub (https://github.com/jk00ANI/Impact‐of‐Fertilization‐on‐Grassland‐Core‐Soil‐Microbiota‐Abundance‐in‐the‐Field‐and‐Growth‐in‐vitro).

### 2.4 Bacterial Strains and Cultivation of Biomarker Bacteria

Since 16S rRNA gene analysis did not provide species‐level resolution, representative species from the identified core genera available in public strain repositories were selected. For further experiments, bacteria were selected based on the following criteria: (i) Culturable type strain, (ii) Biosafety level 1 (iii) isolated from soil if possible. This led to an initial selection of 7 potential biomarker strains (Table S5), which were purchased from public strain repositories and recovered using media and culture conditions recommended by the supplier. Growth of candidate biomarker strains was evaluated on agar plates with seven different media at 30° and 37°C, totaling 14 conditions. Media tested included the following six widely used complex media reported to support the cultivation of a wide range of bacteria: nutrient agar (NA), brain heart infusion (BHI), Reasoner's 2A (R2A), Luria Bertani (LB), tryptic soy agar (TSA) and 2xTY. All media were prepared according to the formulations and protocols provided by the German Collection of Microorganisms and Cell Cultures (DSMZ; https://www.dsmz.de/collection/catalogue/microorganisms/culture‐technology/list‐of‐media‐for‐microorganisms). In addition, growth was tested on a custom‐made soil medium (SM) that was formulated to mimic the nutritional conditions of the study sites (Supporting Information).

Growth of biomarker candidates was tested in liquid media of R2A, NB and SM in microtiter plates using a BioTek Synergy H1M (Supporting Information). The effect of PS on the growth of selected culturable biomarker bacteria was investigated in shake flasks. R2A medium was prepared and PS was added to a final concentration of 2% (v/v), which is an estimation of the amount of PS applied in a single fertilization. To exclude contamination by bacteria present in PS, solids were removed by centrifugation and the remaining supernatant was filter‐sterilized twice prior to addition to R2A medium. Biomarker bacteria were grown to stationary phase in 5 mL of R2A medium in glass tubes. Bacteria were then washed with sterile saline (0.9% (w/v) NaCl in H_2_O) and used to inoculate 50 mL of R2A medium with or without 2% (v/v) PS to a starting OD_600_ of 0.1. The cultures were incubated at 30°C and growth was monitored by measuring OD_600_ until the stationary growth phase was reached. Growth rates and carrying capacity for individual cultures were calculated using the R package Growthcurver v0.3.1 that fits data to a logistic equation (Sprouffske and Wagner 2016). Growth curves were plotted and statistical analysis of growth rates and carrying capacity was performed using GraphPad Prism (Mavrevski et al. 2018).

## 3 Results

### 3.1 Effects of Different Fertilisation Regimes on Diversity of Soil Bacteria in Grassland Ecosystems

**FIGURE 2:**
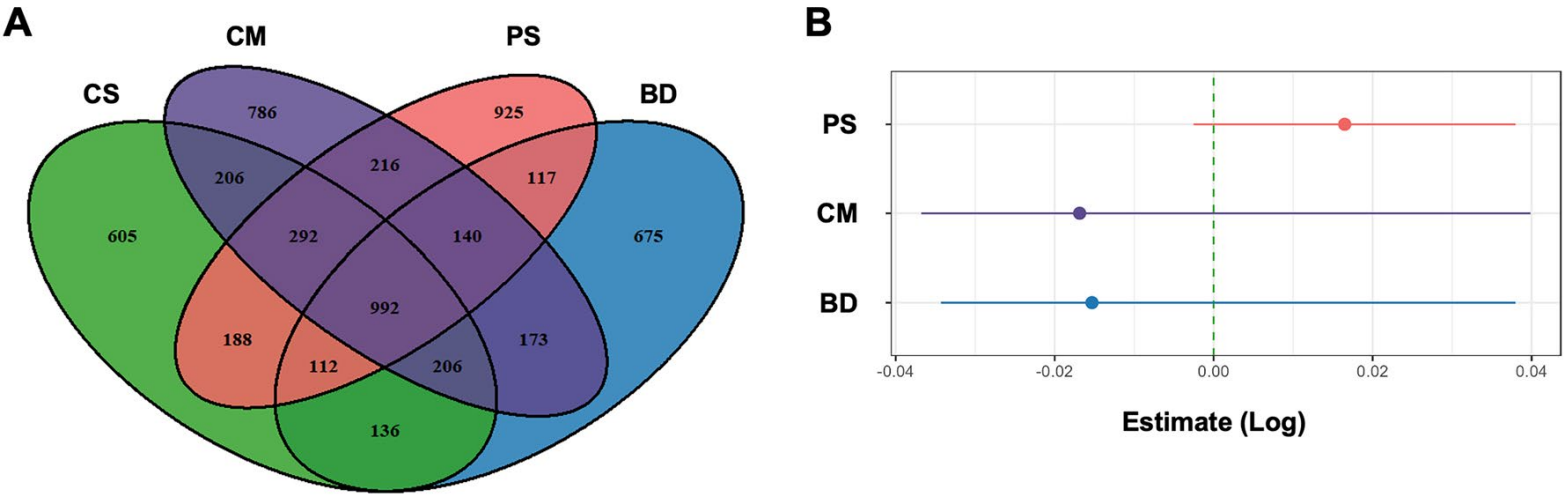
Differences in microbial community composition of grassland soil fertilised with pig slurry (PS), biogas digestate (BD), or cow manure (CM) or soil samples from minimally fertilised control sites (CS). (A) Venn diagram of unique and shared bacterial taxa found in soils fertilised with different organic regimes. (B) Pielou's Evenness indices were calculated using the abundance of the 992 shared ASVs within the different fertilisation regimes. Effects of fertilisation regimes on Pielou's Evenness were tested using generalised linear mixed effect models.

To explore the impact of organic fertilisation on the diversity of bacterial communities in the soil of the grassland ecosystem, we collected soil samples from study sites managed with different fertilisation regimes: pig slurry (PS), cow manure (CM), biogas digestate (BD) and control soil (CS). Following DNA isolation and 16S rRNA gene amplicon sequencing, we obtained a total of 2,303,276 reads that passed the quality criteria. Based on 100% sequence identity, 5769 distinct ASVs were identified, of which 992 were shared among all fertilisation regimes. These 992 ASVs represented 84.7% of all reads in the dataset. 112–292 ASVs were found to be shared by at least two fertilisation regimes, and 605–925 ASVs were unique to a specific regime (Figure 2A). This suggested an uneven distribution of unique and shared taxa among the different fertilisation regimes and a strong representation of the shared bacterial core microbiota.

To analyse the variation in abundance of the 992 shared ASVs, Pielou's Evenness indices (*J*) were compared (Figure 2B) and an overall high evenness in all treatment groups with no significant differences was observed (Table S2).

**FIGURE 3:**
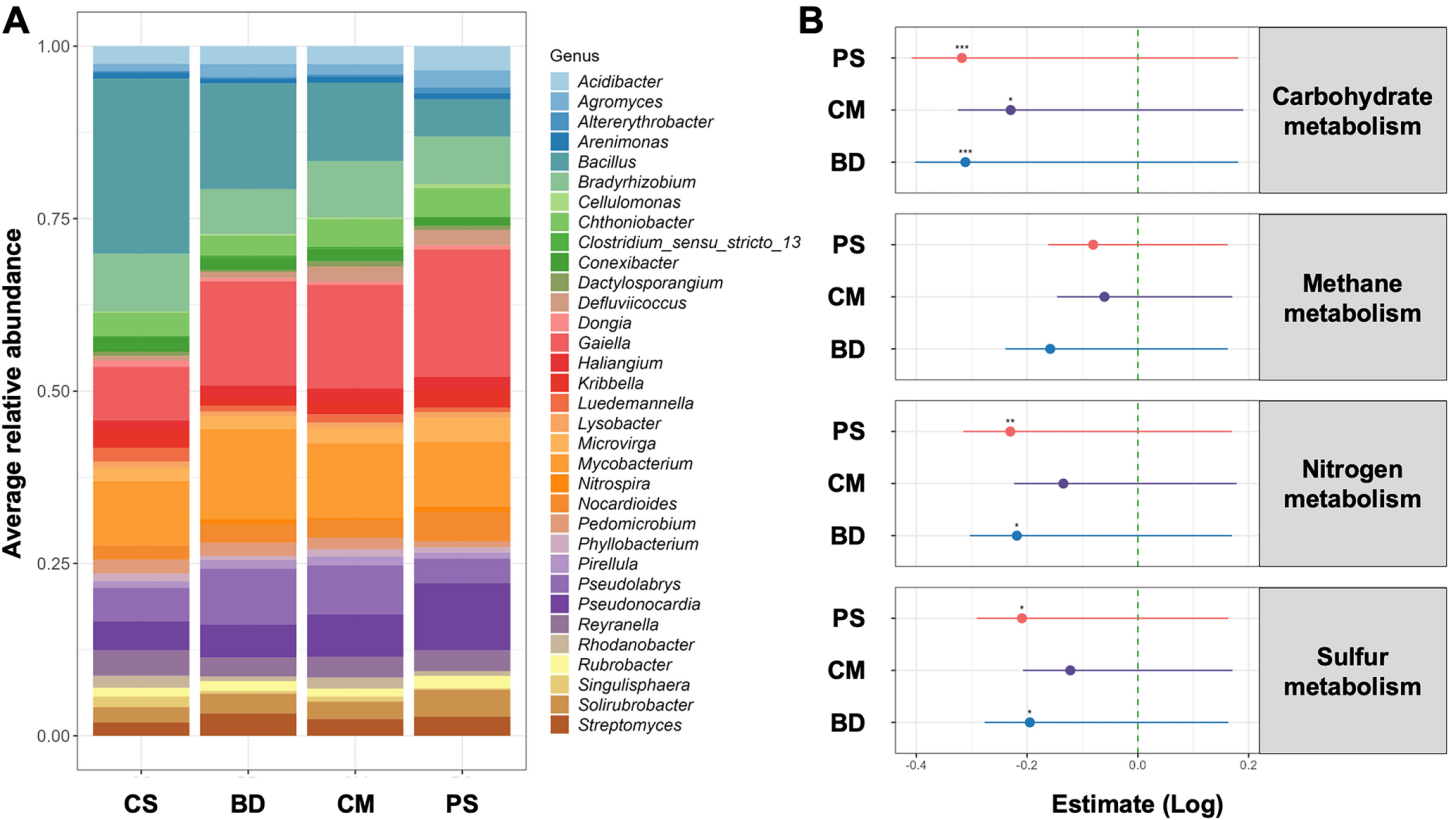
Abundance and functionality of the bacterial core soil microbiota of grasslands under different fertilisation regimes. (A) Community composition of the bacterial soil core microbiota of grasslands fertilised with pig slurry (PS), biogas digestate (BD), or cow manure (CM) or soil samples from minimally fertilised control sites (CS). (B) Comparison of the relative abundance of KEGG orthologs for carbohydrate, methane, nitrogen and sulfur metabolism at study sites of the four fertilisation regimes. Effects of fertilisation regimes on relative abundance of KEGG orthologs were modelled using generalised linear mixed models (*: *p* < 0.05; **: *p* < 0.01; ***: *p* < 0.001).

We next sought to identify the most abundant, known, and culturable representatives of the bacterial core microbiota. To this end, the 992 ASVs of the soil core bacteria were filtered for those that were present in at least 50% of all samples with a relative abundance of at least 0.01%. Moreover, ASVs without valid taxonomic identification at the genus level (unknown or *Candidatus* species/genera) as well as those classified as unculturable were excluded. This resulted in a total of 33 genera representing the validly described core soil bacteria of our data set. These 33 genera showed characteristic patterns of abundance in each of the fertilisation regimes and the differences in abundance patterns were most pronounced between CS and PS‐treated soil (Figure 3A).

To assess if these changes in abundance patterns of the bacterial core microbiota also may have consequences on the functional level, we predicted the KEGG orthologs for carbohydrate, methane, nitrogen and sulfur metabolism associated with ASVs of the 33 core genera across all samples and fertilisation regimes using PICRUSt2 (Douglas et al. 2020). This suggested a substantial reduction in KEGG orthologs of all four functional categories by all fertilisation regimes compared to CS (Figure 3B, Table S3). The predicted reduction in functional orthologs for carbohydrate metabolism was significant for all three fertilisation regimes (BD: β = 0.311, *p* < 0.001; CS: β = −0.230, *p* < 0.05; PS: β = 0.317, *p* < 0.001). KEGG orthologs for nitrogen metabolism were significantly reduced in BD (β = −0.218, *p* < 0.05) and PS (β = −0.230, *p* < 0.01) samples. Similarly, a significant reduction in KEGG orthologs for sulfur metabolism was predicted for BD (β = −0.195, *p* < 0.05) and PS (β = −0.209, *p* < 0.05). KEGG orthologs for methane metabolism were also reduced by all treatments but this effect only was marginally significant in BD samples (β = 0.158, *p* = 0.052).

### 3.2 Microbial Biomarkers of Soil Fertilisation Status

**FIGURE 4:**
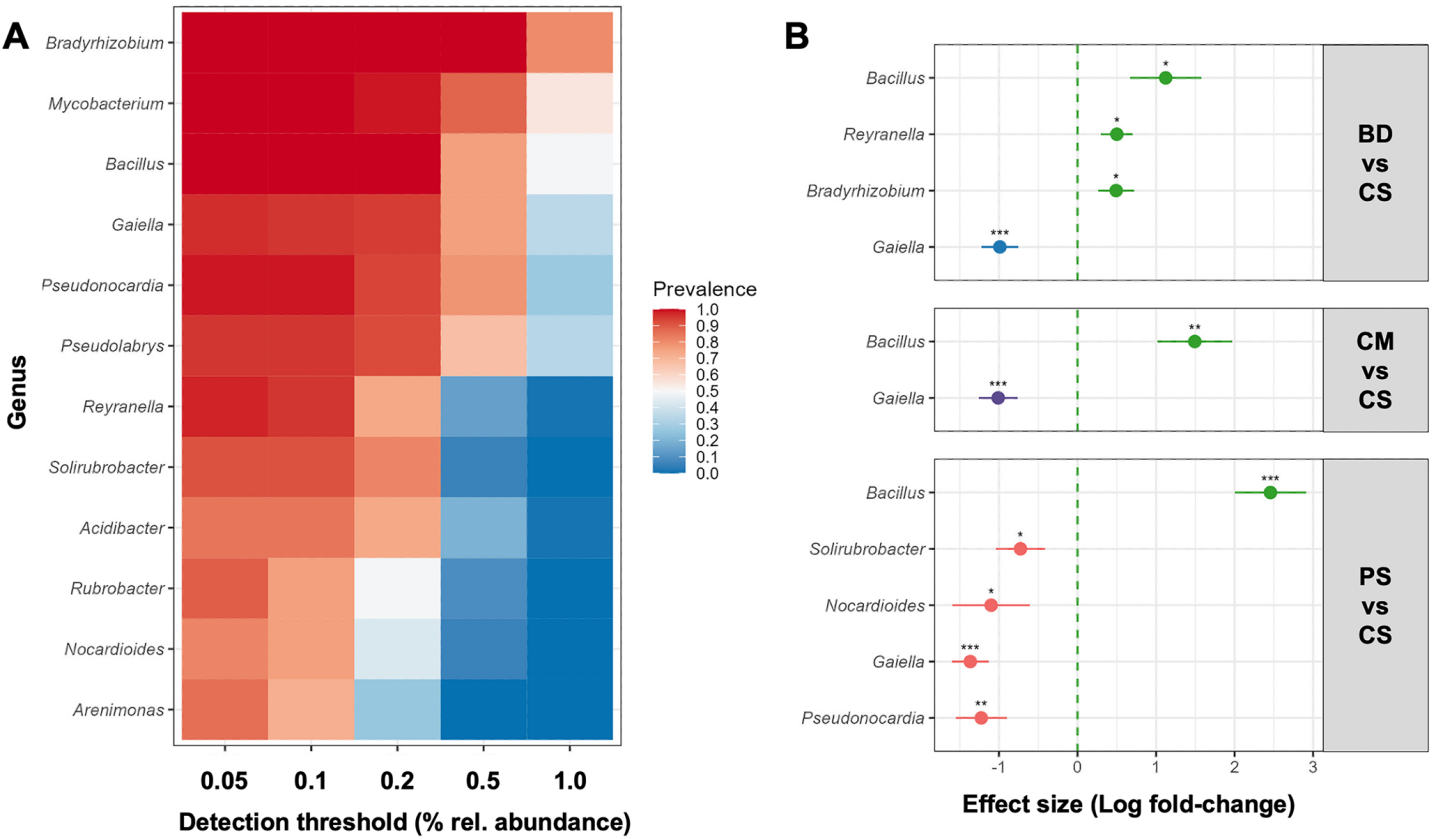
(A) Abundance and prevalence of known bacteria genera across fertilisation regimes. (B) Potential microbial biomarkers in soil of grassland ecosystems of the Swabian Alb indicative of untreated CS soil or fertilisation with BD, CM or PS. Biomarkers were identified by comparing relative abundance of the 12 genera shown in (A) computing effect size using MaAsLin2 (*: *p* < 0.05; **: *p* < 0.01; ***: *p* < 0.001).

Based on the characteristic patterns of abundance of the bacterial core microbiota, we hypothesized that some of these genera may be potential biomarkers of soil fertilisation status. We thus further analysed the genera of the bacterial core microbiota to identify taxa that are representative of the observed differences between fertilisation regimes. To narrow down the list of potential biomarker bacteria, we sorted the 33 known genera according to prevalence at different levels of relative abundance (Figure 4A). Filtering for genera with a prevalence of at least 75% in all samples at a detection threshold of at least 0.05% relative abundance provided a total of 12 known genera, which were used for further analysis.

To identify those bacterial genera that may serve as biomarkers for specific fertilisation regimes, we applied MaAsLin2, a generalised linear mixed model. Estimation of effect size, the generalised linear models of MaAsLin2 allow identification of taxa that most likely explain differences between fertilisation regimes considering their abundance. Significant association with any of the regimes was observed for 7 genera (Figure 4B). The genera *Bacillus*, *Reyranella*, and *Bradyrhizobium* are characteristic of CS sites. *Gaiella* was associated with BD, CM, and PS, and *Nocardioides*, *Solirubrobacter*, and *Pseudocardia* are associated with PS‐fertilised soil.

**FIGURE 5:**
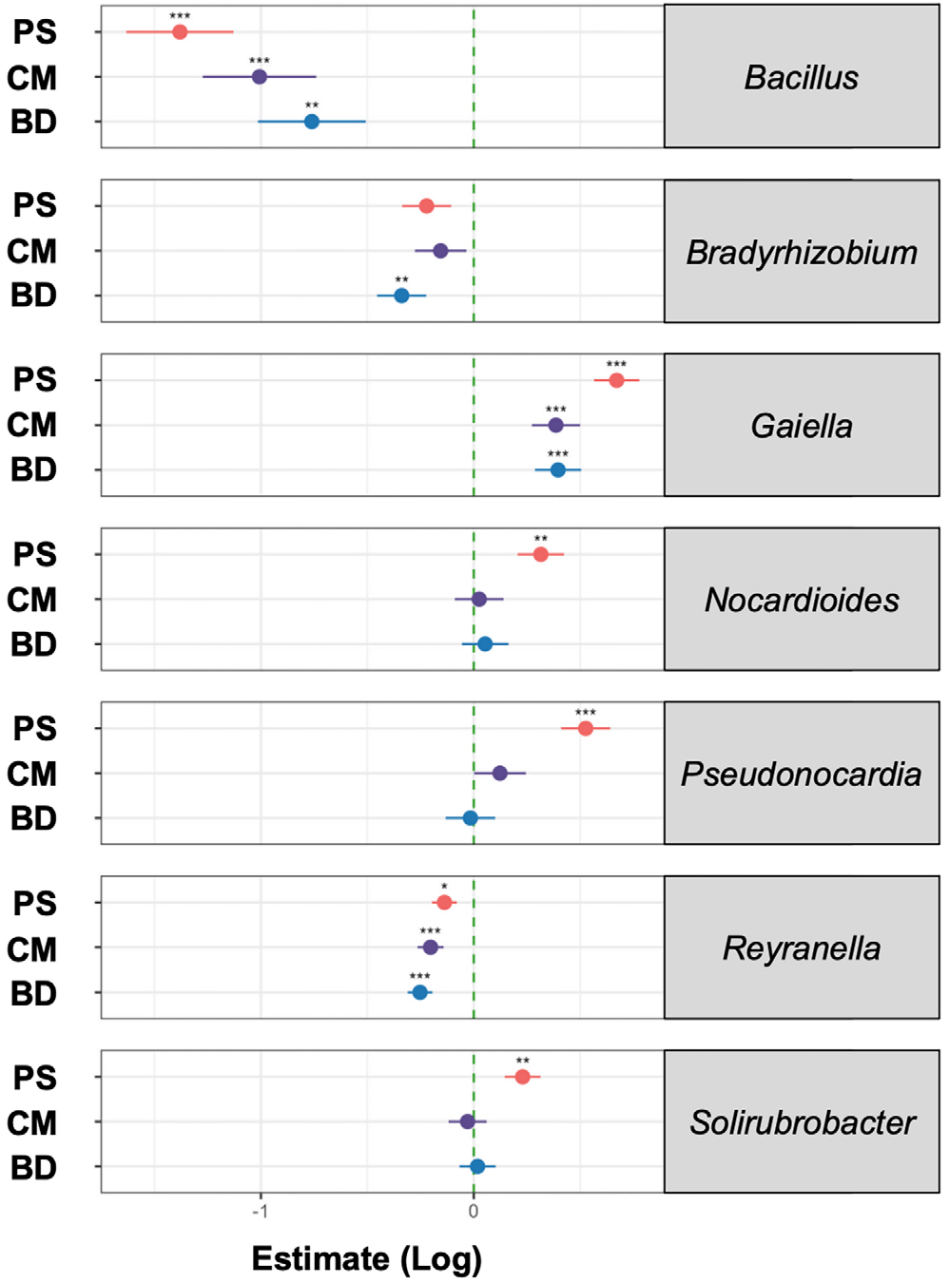
Response of potential bacterial biomarker genera to different fertilisation regimes (PS, BD, CM, and CS) applied to grassland sites in the Swabian Alb. Abundance of biomarker bacteria was transformed prior to analysis and fitted using generalised linear mixed models (GLMMs) with a beta distribution (*: *p* < 0.05; **: *p* < 0.01; ***: *p* < 0.001).

We then compared the relative abundance of the 7 biomarker genera in samples of all fertilisation regimes using a generalised linear mixed model with a beta distribution (log link) (Figure 5, Table S4). CS sites show predominance of *Bacillus* (CS vs. BD: β = −0.761, *p* < 0.01; CS vs. CM: β = −1.006, *p* < 0.001; CS vs. PS: β = −1.38, *p* < 0.001), *Bradyrhizobium* (CS vs. BD: β = −0.338, *p* < 0.01) and *Reyranella* (CS vs. BD: β = −0.252, *p* < 0.001; CS vs. CM: β = −0.203, *p* < 0.001; CS vs. PS: β = −0.137, *p* < 0.05). Genera that were predominant on fertilised sites were *Gaiella* (CS vs. BD: β = 0.396, *p* < 0.001; CS vs. CM: β ~= 0.386, *p* < 0.001; CS vs. PS: β = 0.671, *p* < 0.001), *Nocardioides* (CS vs. PS: β = 0.315, *p* < 0.01), *Pseudonocardia* (CS vs. PS: β = 0.525, *p* < 0.001), and *Solirubrobacter* (CS vs. PS: β = 0.229, *p* < 0.01). This highlights that PS fertilisation has the most profound impact on the abundance of the identified biomarker bacteria.

### 3.3 Culturable Representatives of the Bacterial Core Soil Microbiota

To study the effect of fertilisation on the growth of representative members of core soil bacteria in the lab, we searched public microbial culture collections for type strains of the 7 potential biomarker genera identified by MaAsLin2. For genera with several type strains available, preference was given to strains isolated from soil and, when possible, widely used model strains. We found type strains for all genera (Table S5) but failed to obtain *Reyranella soli* NBRC 108950^T^ even after repeated efforts. Although *Gaiella occulta* CECT 7815^T^ was successfully grown in the lab on its recommended medium (data not shown), this strain was excluded from further experiments due to extremely slow growth. For the remaining five potential biomarker genera, type strains isolated from soil or root nodules were successfully grown in the lab on agar plates of the media recommended by the provider, and these strains were used for further analysis.

To identify conditions of cultivation that support the growth of a maximum number of biomarkers, *Bacillus subtilis* DSM10^T^, *Bradyrhizobium japonicum* DSM 30131^T^, *Nocardioides albus* DSM 43109^T^, *Pseudonocardia antarctica* DSM 44749^T^, and *Solirubrobacter pauli* DSM 14954^T^ were tested on agar plates of six different standard media plus a custom‐made soil medium (SM). Nutrient Agar (NA), Reasoner's 2A (R2A) agar, and SM agar supported the growth of all type strains tested (Table S5) and were thus used to test the growth of all strains in liquid culture. *P. antarctica* showed good growth but formed aggregates in all media (data not shown), which prevented reliable measurement of OD_600_. Aiming for a rapid comparative assessment of growth characteristics of core soil representatives in response to fertilisation, this strain was also excluded from further experiments.

The remaining four potential biomarker bacteria were compared for growth on R2A, nutrient broth (NB) and SM in microtiter plates (Figure S1). This revealed that all three media supported the growth of the selected bacteria to some degree but with strong differences in growth rates and final optical densities between strains and across media. Moreover, considerable variability between individual experiments was noted resulting in high standard deviations of the mean. This was mainly attributed to strong differences in lag phase and was particularly evident for *N. albus* DSM 43109^T^ and *S. pauli* DSM 14954^T^ on SM and NB.

**FIGURE 6:**
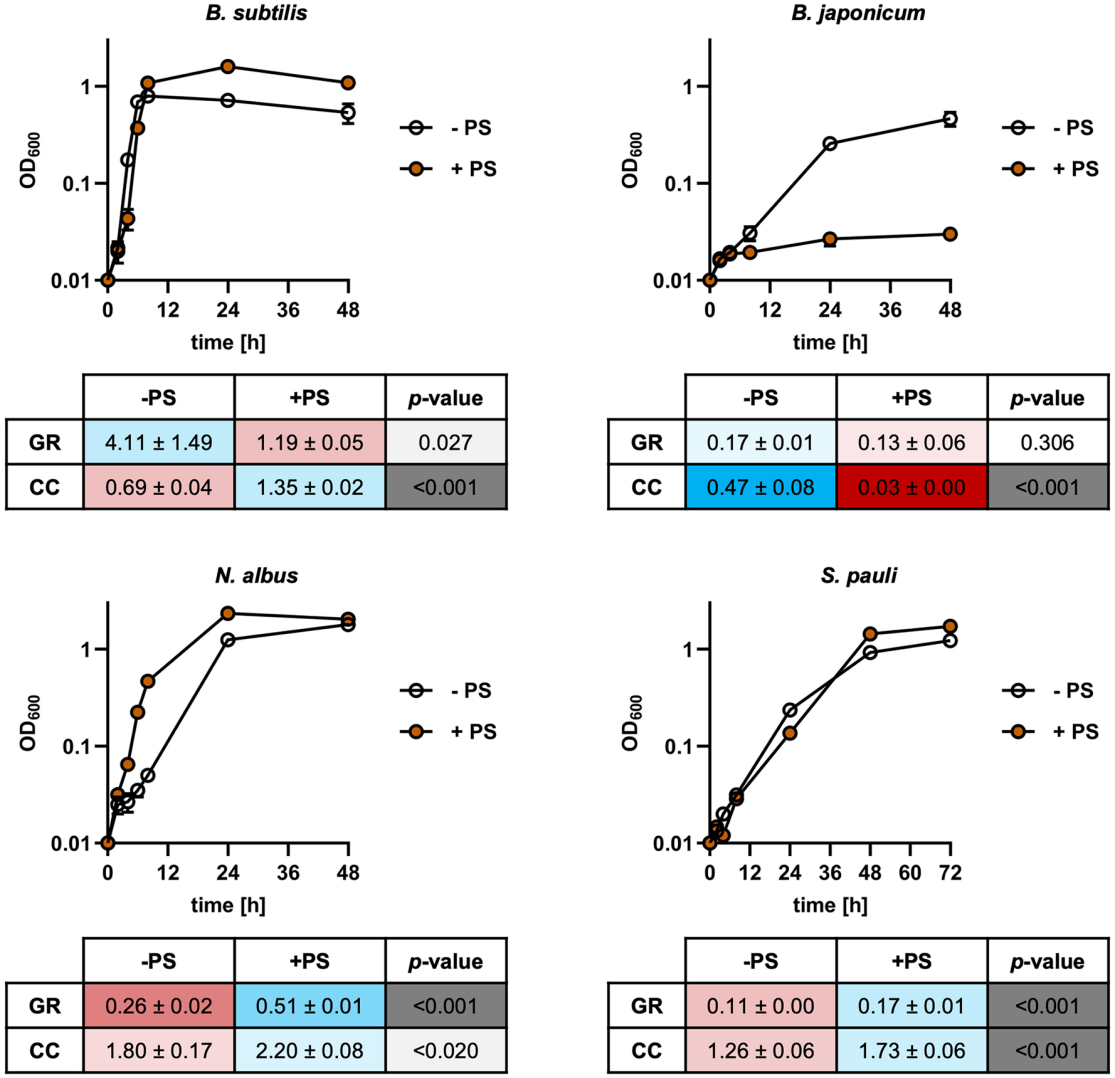
Effect of pig slurry on growth of culturable soil representative bacteria in vitro. All four biomarker bacteria were grown on R2A medium with (filled circles; +PS) or without (open circles; −PS) 2% (v/v) pig slurry. Growth was monitored by measuring OD_600_ at the indicated timepoints. Growth rates (GR) and carrying capacity (CC) for individual cultures were calculated by fitting OD_600_ values to a logistic equation and are plotted below growth curves. All values are mean ± standard deviation of *n* = 3 independent cultures per strain. Statistical analysis was performed by Student's *t*‐test on GR and CC calculated for individual cultures.

Based on these results, R2A medium was the medium that gave the most consistent results for all four strains and selected for a comparative analysis of the effect of PS on the growth of the four selected core soil bacteria in shake flasks (Figure 6). Interestingly, the effects of PS on growth in vitro mirrored abundance patterns observed in the field samples. In line with a reduced abundance in PS‐fertilised soil samples (Figure 5), growth rates and carrying capacity of *B. japonicum* were reduced in the presence of PS and this effect was significant for carrying capacity (Figure 6). Similarly, growth rates of *B. subtilis* were significantly reduced by PS but carrying capacity was, however, increased. Conversely, growth rates and carrying capacity of *N. albus* and *S. pauli* in R2A were significantly increased in the presence of PS (Figure 6) matching their increased abundances in PS‐fertilised soil samples (Figure 5).

**FIGURE 7:**
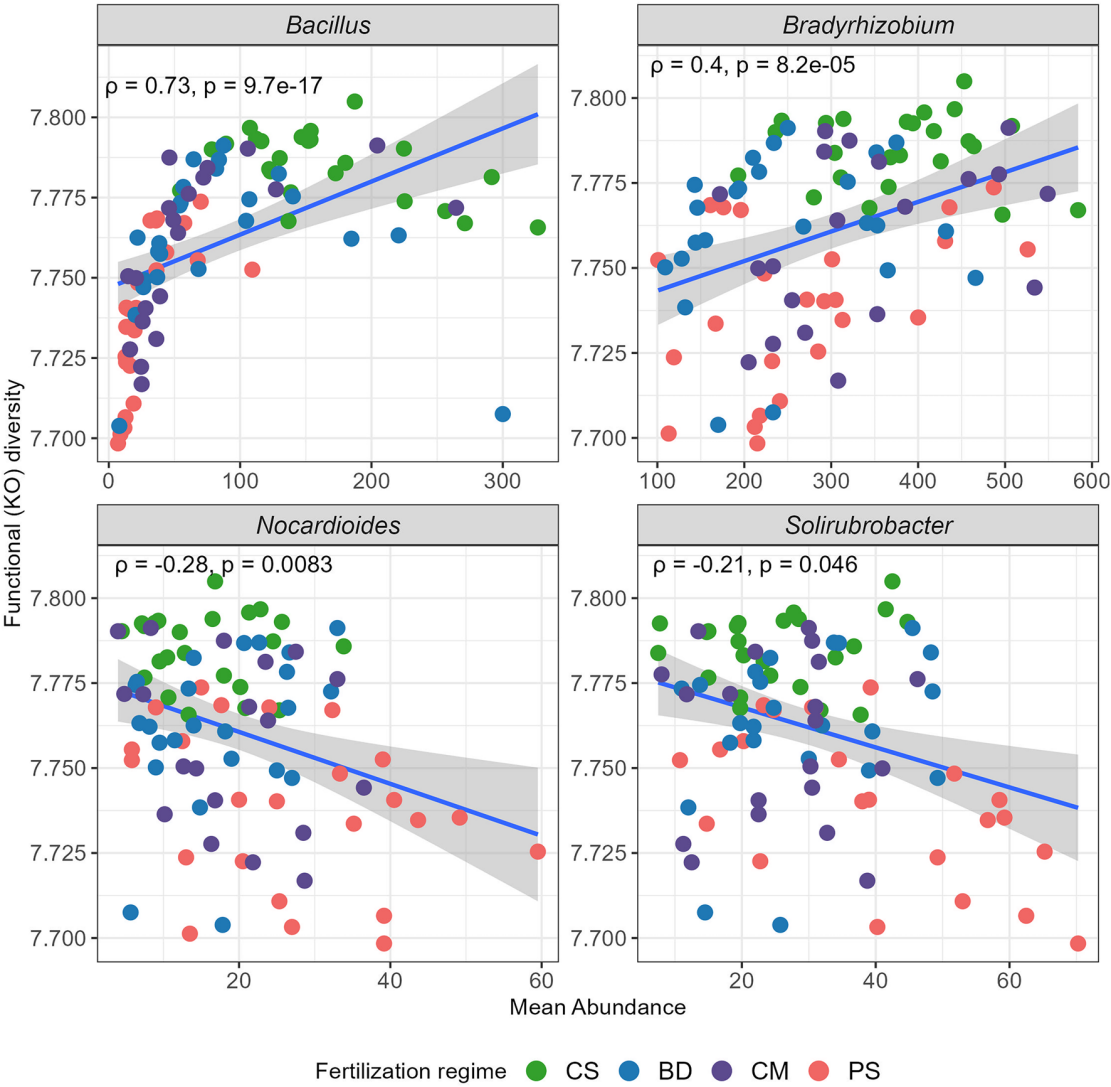
Spearman correlation between the functional diversity (Shannon index) and abundance of the potential bacterial biomarkers of fertilisation. Functional diversity was calculated using PICRUSt2 based on KEGG orthologs in genomes of the database belonging to the ASVs at each study site.

To finally test if there are differences in the functional capacity of the members of the genera *Bacillus*, *Bradyrhizobium*, *Nocardioides*, and *Solirubrobacter* depending on the fertilisation regime we again performed PICRUSt2 analyses. Using KEGG orthologs inferred from ASVs we calculated the functional diversity (Shannon indices) in each sample for the four genera separately. Analysing functional diversity vs. relative abundance (Figure 7; Table S6) revealed a significant positive correlation for the genera *Bacillus* (ρ = 0.73, *p* = 9.7 × 10^−17^) and *Bradyrhizobium* (ρ = 0.4, *p* = 8.2 × 10^−5^) indicating that higher abundance is associated with higher (predicted) functional diversity. Conversely, a significant negative correlation was observed for *Nocardioides* (ρ = −0.28, *p* = 0.0083) and *Solirubrobacter* (ρ = −0.21, *p* = 0.046) suggesting that the higher the abundance of these genera at a fertilised site is, the lower is their (predicted) functional diversity. Moreover, *Bacillus* and *Bradyrhizobium* appeared to be less abundant on PS‐fertilised sites and these communities also were predicted to have lower functional diversity. By contrast, *Nocardioides* and *Solirubrobacter* showed higher abundance on CS‐fertilised study sites and these bacteria were predicted to have a higher functional diversity.

## 4 Discussion

In this study, we examined the impact of organic fertilisation regimes on the bacterial soil microbiome of grassland ecosystems in the Swabian Alb. Our aim was to identify culturable representatives of the bacterial core microbiota as biomarkers of soil fertilisation status, which could be used to investigate the effects of fertilisation and other anthropogenic factors on microbial growth in vitro. Using high‐throughput 16S rRNA gene amplicon sequencing, we identified 5769 distinct ASVs in a total of 2,303,276 reads. These numbers are in accordance with other studies on the soil microbiome. A recent study assessing the effect of chemical fertilisation on soil microbial diversity in a controlled setting with pot‐grown wheat plants found 10,298 ASVs in 5.3 million reads of 286 samples (Reid et al. 2024). A large survey on microbial diversity of soil of different vegetation types across Europe found on average 8000–8300 bacterial OTUs in soil samples of extensively and intensively managed grasslands, respectively (Labouyrie et al. 2023).

Approx. 85% of sequence reads in our data set belong to taxa shared by all fertilisation regimes. Our sequencing data suggested that organic fertilisation had a profound impact on the structure of the core bacterial soil microbiome and these effects were most pronounced in study sites fertilised with PS. Filtering shared ASVs for highly abundant and prevalent taxa with validly described and culturable representatives identified 12 potential biomarker genera, which were further analysed using MaAsLin2, a common method to identify biomarkers indicative of specific conditions in microbiome data (Mallick et al. 2021). Additionally, the functionality of these potential biomarker genera was inferred using PICRUSt2‐based predictions. We observed asignificant reduction in KEGG orthologs related to carbohydrate, nitrogen, and sulfur metabolisms indicating that fertilisation regime may disrupt key soil microbial function and consequently influence nutrient cycling. Soil microbes play crucial roles in breaking down organic matter, fixing nitrogen and processing carbon storage with important consequences for ecosystem functions such as soil fertility and plant productivity (Fierer 2017; Delgado‐Baquerizo et al. 2020; Yadav et al. 2021). While our study provides important indications for functional change under organic fertilisation, further research is needed to explore the mechanism, by which a specific treatment influences soil microbial function.

Type strain of potential biomarker genera were obtained from culture collections and, based on their ability to grow on standard media, four species ( *B. subtilis* , *B. japonicum* , *N. albus* , and *S. pauli* ) were selected to investigate the effect of pig slurry on growth in vitro. Results of growth experiments with these four biomarkers reflected their abundance in the field and are mostly in line with their classification according to their hypothesized life strategies. According to the oligotroph‐copiotroph concept, oligotrophic bacteria that grow slowly under apparent optimal conditions are more prevalent in environments with low availability of nutrients whereas copiotrophs are fast‐growing organisms common in high‐nutrient environments (Koch 2001; Ho et al. 2017). These differences in life strategy may also help explain the relative growth rate and hence population size of bacterial groups in a given habitat. *Solirubrobacter* and *Nocardioides* showed higher abundance in PS‐fertilised study sites. Additionally, growth rates and final biomass of *N. albus* and *S. pauli* were increased in the presence of PS. Both genera belong to the phylum *Actinomycetota*, which contains mostly copiotrophic organisms (Stone et al. 2023). Thus, increased growth rates in the presence of higher levels of nutrients may, at least in part, explain the higher abundance of these organisms on PS‐fertilised soil.

According to the MaAsLin2 analysis, *Bacillus* is a biomarker for CS samples, which were obtained from minimally fertilised sites, and the abundance of ASVs belonging to this genus was significantly reduced in the soil of PS‐, CM‐ and BD‐treated sites. This contradicts the classification of the genus *Bacillus* as copiotrophs (Stone et al. 2023). Recently, Xu et al. examined the factors that affect the abundance of *Bacillus subtilis* group species in soil and found higher abundance in grassland soil compared to forest soil (Xu et al. 2024). At the same time, forest soils had higher organic content and less acidic pH and both factors correlated with abundance patterns. Nutrient availability is one of the major drivers shaping the soil microbiome (Fierer 2017) and organic fertilisers are a rich source of nutrients including organic carbon, nitrogen and phosphorous (Marinari et al. 2000; Plaza et al. 2007). Thus, the introduction of organic content by fertilisation might explain the reduced abundance of the genus *Bacillus* under all three fertilisation regimes in our study. Of note, *B. subtilis* DSM10 displayed reduced growth rates but higher final OD_600_ when grown in vitro in the presence of PS. While reduced growth rates would actually be in line with reduced abundance in conditions with higher nutrient availability, accumulation to higher biomass somehow contradicts the observations from the field. However, higher abundance of the genus *Bacillus* in low nutrient grassland soil was found to occur together with other genera of the *Bacillota* phylum in strongly interconnected modules (Xu et al. 2024), which may indicate cooperation. Such cooperative interactions as well as potential antagonistic interactions in the field, in which one member of the consortium inhibits another, cannot be modelled by single strain cultivations in vitro.

As members of the Alphaproteobacteria, the genus *Bradyrhizobium* belongs to the oligotrophic organisms associated with low‐nutrient environments (Stone et al. 2023). In line with this classification, *Bradyrhizobium* was suggested by MaAsLin2 as another biomarker genus indicative of untreated soil samples and the abundance of ASVs was significantly reduced in BD‐fertilised sites. Similar to our results, a lower abundance of bacterial nitrogen‐fixers was observed in intensively vs. extensively used grasslands in a large survey of microbial diversity in soils across Europe (Labouyrie et al. 2023). *B. japonicum* is a member of the *Rhizobiaceae* family of bacterial symbionts that are actively recruited by legume plants into specialised root nodules for nitrogen fixation (Hungria et al. 2015). Recruitment of nitrogen‐fixing bacteria by plants is mediated by root exudates that are regulated by environmental stimuli (Canarini et al. 2019; Trivedi et al. 2020), shape the composition of the soil microbiome (Seitz et al. 2022), and may have a profound impact on the nitrogen cycle in soil (Coskun et al. 2017). Thus, the reduced abundance of ASVs belonging to the genus *Bradyrhizobium* in fertilised soil could be explained by a down‐regulation of exudates by plants in response to nitrogen influx in reduced numbers of nitrogen‐fixing bacteria. Our in vitro growth experiments revealed that PS strongly inhibited the growth of *B. japonicum* . It is thus possible that the reduction of *Bradyrhizobium* spp. in fertilised soil may be a direct consequence of growth inhibition by organic fertilisers.

In summary, our study provides culture‐independent and ‐dependent evidence of the impact of organic fertilisation on the structure of bacterial soil microbiomes. Using a large 16S rRNA gene amplicon data set obtained from field samples, we were able to show that a large portion of the observed taxa is shared across all sampling sites and define the core microbiota of known bacterial genera. This bacterial core microbiota responded to different organic fertilisation regimes by characteristic changes in abundance patterns. Building on core‐microbiome and differential abundance, MaAsLin2 analysis allowed identification of biomarker genera that show characteristic differences in abundance on PS‐fertilised and CS sites. The effects of PS on the growth of culturable representatives of these biomarker genera are in line with their observed changes in abundance in field samples. Thus, further in vitro experiments may provide a tool to assess the impact of individual parameters of organic fertilisation or other environmental factors on members of the bacterial core soil microbiota. Given the high genetic variability even within one species and, even more so, on the genus level, results obtained with type strains in in vitro growth experiments are somewhat speculative and only a first indication of the effects of organic fertilisation on individual components of the bacterial core microbiome. Consequently, the presented in vitro systems and the findings need to be validated in further experiments with for example, primary soil isolates and under more natural conditions reflecting temperature fluctuations.

## Author Contributions

**Rostand R. Chamedjeu:** investigation, formal analysis, writing – original draft, writing – review and editing. **Kunal Jani:** methodology, investigation, formal analysis, validation, writing – review and editing, data curation. **Karoline Jetter:** investigation, formal analysis, writing – review and editing. **Kerstin Wilhelm:** investigation, methodology, writing – review and editing. **Patrick Schäfer:** conceptualization, funding acquisition, writing – review and editing, supervision, resources. **Lena Wilfert:** conceptualization, funding acquisition, writing – review and editing, formal analysis, supervision, resources. **Simone Sommer:** conceptualization, funding acquisition, formal analysis, writing – review and editing, supervision, resources. **Christian U. Riedel:** conceptualization, funding acquisition, validation, writing – original draft, writing – review and editing, supervision, resources.

## Conflicts of Interest

The authors declare no conflicts of interest.

## Supporting information


**Data S1:** emi470235‐sup‐0001‐supinfo.pdf.

## Data Availability

This work is based on data collected during the IMPALA project in cooperation with the Biodiversity Exploratories program (DFG Priority Program 1374). Raw reads of the 16S rRNA gene amplicon sequencing are publicly available on the NCBI database (BioProject: PRJNA1188490, accessible to reviewers under the following link https://dataview.ncbi.nlm.nih.gov/object/SRR31410284?reviewer=uvlb4flva3irladhfldd8i4ioi) and on the Biodiversity Exploratories Information System (http://doi.org/10.17616/R32P9Q). The datasets are listed in the reference section (Jetter et al. 2025).

## References

[emi470235-bib-0001] Babin, D. , C. Leoni , A. L. Neal , A. Sessitsch , and K. Smalla . 2021. “Editorial to the Thematic Topic Towards a More Sustainable Agriculture Through Managing Soil Microbiomes.” FEMS Microbiology Ecology 97: fiab094. 34263312 10.1093/femsec/fiab094

[emi470235-bib-0002] Berg, G. , D. Rybakova , D. Fischer , et al. 2020. “Microbiome Definition Re‐Visited: Old Concepts and New Challenges.” Microbiome 8: 103. 32605663 10.1186/s40168-020-00875-0 PMC7329523

[emi470235-bib-0003] Bissett, A. , A. E. Richardson , G. Baker , and P. H. Thrall . 2011. “Long‐Term Land Use Effects on Soil Microbial Community Structure and Function.” Applied Soil Ecology 51: 66–78.

[emi470235-bib-0004] Bohan, D. A. , C. Vacher , A. Tamaddoni‐Nezhad , A. Raybould , A. J. Dumbrell , and G. Woodward . 2017. “Next‐Generation Global Biomonitoring: Large‐Scale, Automated Reconstruction of Ecological Networks.” Trends in Ecology & Evolution 32: 477–487. 28359573 10.1016/j.tree.2017.03.001

[emi470235-bib-0005] Bolyen, E. , J. R. Rideout , M. R. Dillon , et al. 2019. “Reproducible, Interactive, Scalable and Extensible Microbiome Data Science Using QIIME 2.” Nature Biotechnology 37: 852–857. 10.1038/s41587-019-0209-9 PMC7015180 31341288

[emi470235-bib-0006] Callahan, B. J. , P. J. McMurdie , M. J. Rosen , A. W. Han , A. J. A. Johnson , and S. P. Holmes . 2016. “DADA2: High‐Resolution Sample Inference From Illumina Amplicon Data.” Nature Methods 13: 581–583. 27214047 10.1038/nmeth.3869 PMC4927377

[emi470235-bib-0007] Canarini, A. , C. Kaiser , A. Merchant , A. Richter , and W. Wanek . 2019. “Root Exudation of Primary Metabolites: Mechanisms and Their Roles in Plant Responses to Environmental Stimuli.” Frontiers in Plant Science 10: 157. 30881364 10.3389/fpls.2019.00157 PMC6407669

[emi470235-bib-0008] Caporaso, J. G. , C. L. Lauber , W. A. Walters , et al. 2011. “Global Patterns of 16S rRNA Diversity at a Depth of Millions of Sequences per Sample.” Proceedings of the National Academy of Sciences 108: 4516–4522. 10.1073/pnas.1000080107 PMC3063599 20534432

[emi470235-bib-0010] Chen, H. , and P. C. Boutros . 2011. “VennDiagram: A Package for the Generation of Highly‐Customizable Venn and Euler Diagrams in R.” BMC Bioinformatics 12: 35. 21269502 10.1186/1471-2105-12-35 PMC3041657

[emi470235-bib-0011] Congreves, K. A. , D. C. Hooker , A. Hayes , E. A. Verhallen , and L. L. Van Eerd . 2017. “Interaction of Long‐Term Nitrogen Fertilizer Application, Crop Rotation, and Tillage System on Soil Carbon and Nitrogen Dynamics.” Plant and Soil 410: 113–127.

[emi470235-bib-0013] Cordier, T. , L. Alonso‐Sáez , L. Apothéloz‐Perret‐Gentil , et al. 2021. “Ecosystems Monitoring Powered by Environmental Genomics: A Review of Current Strategies With an Implementation Roadmap.” Molecular Ecology 30: 2937–2958. 32416615 10.1111/mec.15472 PMC8358956

[emi470235-bib-0012] Cordier, T. , A. Lanzén , L. Apothéloz‐Perret‐Gentil , T. Stoeck , and J. Pawlowski . 2019. “Embracing Environmental Genomics and Machine Learning for Routine Biomonitoring.” Trends in Microbiology 27: 387–397. 30554770 10.1016/j.tim.2018.10.012

[emi470235-bib-0014] Coskun, D. , D. T. Britto , W. Shi , and H. J. Kronzucker . 2017. “How Plant Root Exudates Shape the Nitrogen Cycle.” Trends in Plant Science 22: 661–673. 28601419 10.1016/j.tplants.2017.05.004

[emi470235-bib-0009] de Castro, A. P. , G. da R. Fernandes , and O. L. Franco . 2014. “Insights Into Novel Antimicrobial Compounds and Antibiotic Resistance Genes From Soil Metagenomes.” Frontiers in Microbiology 5: 489. 25278933 10.3389/fmicb.2014.00489 PMC4166954

[emi470235-bib-0015] Delgado‐Baquerizo, M. , P. B. Reich , C. Trivedi , et al. 2020. “Multiple Elements of Soil Biodiversity Drive Ecosystem Functions Across Biomes.” Nature Ecology & Evolution 4: 210–220. 32015427 10.1038/s41559-019-1084-y

[emi470235-bib-0016] Dixon, P. 2003. “VEGAN, a Package of R Functions for Community Ecology.” Journal of Vegetation Science 14: 927–930.

[emi470235-bib-0017] Douglas, G. M. , V. J. Maffei , J. R. Zaneveld , et al. 2020. “PICRUSt2 for Prediction of Metagenome Functions.” Nature Biotechnology 38: 685–688. 10.1038/s41587-020-0548-6 PMC7365738 32483366

[emi470235-bib-0018] Fackelmann, G. , M. A. F. Gillingham , J. Schmid , et al. 2021. “Human Encroachment Into Wildlife Gut Microbiomes.” Communications Biology 4: 1–11. 34172822 10.1038/s42003-021-02315-7 PMC8233340

[emi470235-bib-0019] Fierer, N. 2017. “Embracing the Unknown: Disentangling the Complexities of the Soil Microbiome.” Nature Reviews. Microbiology 15: 579–590. 28824177 10.1038/nrmicro.2017.87

[emi470235-bib-0052] Gerhardt, A. 2002. “Bioindicator Species and Their Use in Biomonitoring.” In Environmental Monitoring, 77–123. Encyclopedia of Life Support Systems (EOLSS) Oxford.

[emi470235-bib-0020] Gu, Z. , R. Eils , and M. Schlesner . 2016. “Complex Heatmaps Reveal Patterns and Correlations in Multidimensional Genomic Data.” Bioinformatics 32: 2847–2849. 27207943 10.1093/bioinformatics/btw313

[emi470235-bib-0021] Ho, A. , D. P. Di Lonardo , and P. L. E. Bodelier . 2017. “Revisiting Life Strategy Concepts in Environmental Microbial Ecology.” FEMS Microbiology Ecology 93: fix006. 10.1093/femsec/fix006 28115400

[emi470235-bib-0022] Hungria, M. , P. Menna , and J. R. M. Delamuta . 2015. “Bradyrhizobium, the Ancestor of all Rhizobia: Phylogeny of Housekeeping and Nitrogen‐Fixation Genes.” In Biological Nitrogen Fixation, 191–202. John Wiley & Sons, Ltd.

[emi470235-bib-0023] Hurni, H. , M. Giger , H. Liniger , et al. 2015. “Soils, Agriculture and Food Security: The Interplay Between Ecosystem Functioning and Human Well‐Being.” Current Opinion in Environmental Sustainability 15: 25–34.

[emi470235-bib-0024] Jetter, K. , K. Jani , and R. Chamedjeu . 2025. “16S Microbiome Data From Grassland Sites on Schwäbische Alb.”

[emi470235-bib-0025] Koch, A. L. 2001. “Oligotrophs Versus Copiotrophs.” BioEssays 23: 657–661. 11462219 10.1002/bies.1091

[emi470235-bib-0026] Kopittke, P. M. , B. Minasny , E. Pendall , C. Rumpel , and B. A. McKenna . 2024. “Healthy Soil for Healthy Humans and a Healthy Planet.” Critical Reviews in Environmental Science and Technology 54: 210–221.

[emi470235-bib-0027] Labouyrie, M. , C. Ballabio , F. Romero , et al. 2023. “Patterns in Soil Microbial Diversity Across Europe.” Nature Communications 14: 3311. 10.1038/s41467-023-37937-4 PMC10250377 37291086

[emi470235-bib-0028] Lehmann, J. , D. A. Bossio , I. Kögel‐Knabner , and M. C. Rillig . 2020. “The Concept and Future Prospects of Soil Health.” Nature Reviews Earth and Environment 1: 544–553. 10.1038/s43017-020-0080-8 PMC7116140 33015639

[emi470235-bib-0029] Ma, B. , C. Lu , Y. Wang , et al. 2023. “A Genomic Catalogue of Soil Microbiomes Boosts Mining of Biodiversity and Genetic Resources.” Nature Communications 14: 7318. 10.1038/s41467-023-43000-z PMC10640626 37951952

[emi470235-bib-0030] Mallick, H. , A. Rahnavard , L. J. McIver , et al. 2021. “Multivariable Association Discovery in Population‐Scale Meta‐Omics Studies.” PLoS Computational Biology 17: e1009442. 34784344 10.1371/journal.pcbi.1009442 PMC8714082

[emi470235-bib-0031] Marinari, S. , G. Masciandaro , B. Ceccanti , and S. Grego . 2000. “Influence of Organic and Mineral Fertilisers on Soil Biological and Physical Properties.” Bioresource Technology 72: 9–17.

[emi470235-bib-0032] Matson, P. A. , W. J. Parton , A. G. Power , and M. J. Swift . 1997. “Agricultural Intensification and Ecosystem Properties.” Science 277: 504–509. 20662149 10.1126/science.277.5325.504

[emi470235-bib-0033] Mavrevski, R. , M. Traykov , I. Trenchev , and M. Trencheva . 2018. “Approaches to Modeling of Biological Experimental Data With GraphPad Prism Software.” WSEAS Transactions on Systems and Control Archive 13.

[emi470235-bib-0034] McMurdie, P. J. , and S. Holmes . 2013. “Phyloseq: An R Package for Reproducible Interactive Analysis and Graphics of Microbiome Census Data.” PLoS One 8: e61217. 23630581 10.1371/journal.pone.0061217 PMC3632530

[emi470235-bib-0035] Philippot, L. , C. Chenu , A. Kappler , M. C. Rillig , and N. Fierer . 2024. “The Interplay Between Microbial Communities and Soil Properties.” Nature Reviews. Microbiology 22: 226–239. 37863969 10.1038/s41579-023-00980-5

[emi470235-bib-0036] Plaza, C. , J. C. García‐Gil , and A. Polo . 2007. “Microbial Activity in Pig Slurry‐Amended Soils Under Aerobic Incubation.” Biodegradation 18: 159–165. 16758274 10.1007/s10532-006-9051-0

[emi470235-bib-0037] R Core Team . 2022. “R: A Language and Environment for Statistical Computing. R Foundation for Statistical Computing.”

[emi470235-bib-0038] Raupp, P. , Y. Carrillo , and U. N. Nielsen . 2024. “Soil Health to Enhance Ecological Restoration and Conservation.” Journal of Sustainable Agriculture and Environment 3: e70022.

[emi470235-bib-0039] Reid, T. E. , V. N. Kavamura , A. Torres‐Ballesteros , et al. 2024. “Agricultural Intensification Reduces Selection of Putative Plant Growth‐Promoting Rhizobacteria in Wheat.” ISME Journal 18: wrae131. 38990206 10.1093/ismejo/wrae131 PMC11292143

[emi470235-bib-0040] Seitz, V. A. , B. B. McGivern , R. A. Daly , et al. 2022. “Variation in Root Exudate Composition Influences Soil Microbiome Membership and Function.” Applied and Environmental Microbiology 88: e0022622. 35536051 10.1128/aem.00226-22 PMC9195941

[emi470235-bib-0041] Siebert, J. , M. P. Thakur , T. Reitz , et al. 2019. “Chapter Two ‐ Extensive Grassland‐Use Sustains High Levels of Soil Biological Activity, but Does Not Alleviate Detrimental Climate Change Effects.” In Advances in Ecological Research. Resilience in Complex Socio‐Ecological Systems, edited by D. A. Bohan and A. J. Dumbrell , 25–58. Academic Press.

[emi470235-bib-0042] Sprouffske, K. , and A. Wagner . 2016. “Growthcurver: An R Package for Obtaining Interpretable Metrics From Microbial Growth Curves.” BMC Bioinformatics 17: 172. 27094401 10.1186/s12859-016-1016-7 PMC4837600

[emi470235-bib-0043] Stone, B. W. G. , P. Dijkstra , B. K. Finley , et al. 2023. “Life History Strategies Among Soil Bacteria‐Dichotomy for Few, Continuum for Many.” ISME Journal 17: 611–619. 36732614 10.1038/s41396-022-01354-0 PMC10030646

[emi470235-bib-0044] Thakur, M. P. , H. R. P. Phillips , U. Brose , et al. 2020. “Towards an Integrative Understanding of Soil Biodiversity.” Biological Reviews of the Cambridge Philosophical Society 95: 350–364. 31729831 10.1111/brv.12567 PMC7078968

[emi470235-bib-0045] Timmis, K. , and J. L. Ramos . 2021. “The Soil Crisis: The Need to Treat as a Global Health Problem and the Pivotal Role of Microbes in Prophylaxis and Therapy.” Microbial Biotechnology 14: 769–797. 33751840 10.1111/1751-7915.13771 PMC8085983

[emi470235-bib-0046] Trivedi, P. , B. D. Batista , K. E. Bazany , and B. K. Singh . 2022. “Plant‐Microbiome Interactions Under a Changing World: Responses, Consequences and Perspectives.” New Phytologist 234: 1951–1959. 35118660 10.1111/nph.18016

[emi470235-bib-0047] Trivedi, P. , J. E. Leach , S. G. Tringe , T. Sa , and B. K. Singh . 2020. “Plant‐Microbiome Interactions: From Community Assembly to Plant Health.” Nature Reviews. Microbiology 18: 607–621. 32788714 10.1038/s41579-020-0412-1

[emi470235-bib-0048] Wood, S. A. , and J. C. Blankinship . 2022. “Making Soil Health Science Practical: Guiding Research for Agronomic and Environmental Benefits.” Soil Biology and Biochemistry 172: 108776.

[emi470235-bib-0049] Xu, X. , A. Pioppi , H. T. Kiesewalter , M. L. Strube , and Á. T. Kovács . 2024. “Disentangling the Factors Defining *Bacillus Subtilis* Group Species Abundance in Natural Soils.” Environmental Microbiology 26: e16693. 39324517 10.1111/1462-2920.16693

[emi470235-bib-0050] Yadav, A. N. , D. Kour , T. Kaur , et al. 2021. “Biodiversity, and Biotechnological Contribution of Beneficial Soil Microbiomes for Nutrient Cycling, Plant Growth Improvement and Nutrient Uptake.” Biocatalysis and Agricultural Biotechnology 33: 102009.

[emi470235-bib-0051] Zhang, Z. , L. Zhang , L. Zhang , H. Chu , J. Zhou , and F. Ju . 2024. “Diversity and Distribution of Biosynthetic Gene Clusters in Agricultural Soil Microbiomes.” mSystems 9: e0126323. 38470142 10.1128/msystems.01263-23 PMC11019929

