# Supplementary material for: Impact of Fertilisation on the Bacterial Core Microbiome of Grassland Soils: Abundance in the Field and Growth In Vitro
Source: Environ Microbiol Rep. 2025 Nov 21;17(6):e70235. doi: 10.1111/1758-2229.70235 (PMC12635495; doi:10.1111/1758-2229.70235)
Supplement: Supplementary file 1 — Data S1: emi470235‐sup‐0001‐supinfo.pdf. [file EMI4-17-e70235-s001.pdf]

Supplementary Data

Impact of Fertilization on the Bacterial Core Soil Microbiome of Grassland Soils: Abundance in the Field and Growth *in vitro*

Rostand R. Chamedjeu[1,2,#], Kunal Jani[2,#], Karoline Jetter[1,2], Kerstin Wilhelm[2], Patrick Schäfer[3], Lena Wilfert[2], Simone Sommer[2], and Christian U. Riedel[1,2,*]

[1] Microbial Biotechnology, Department of Biology, University of Ulm, Ulm, Germany

[2] Institute of Evolutionary Ecology and Conservation Genomics, University of Ulm, Germany

[3] Institute of Phytopathology, Research Centre for BioSystems, Land Use and Nutrition, Justus Liebig University, Germany

[#] equal contribution

[*] **Correspondence:** christian.riedel@uni-ulm.de; ORCID: 0000-0001-7134-7085

Keywords: Grassland ecosystems, organic fertilization, 16S rRNA gene amplicon sequencing, soil microbiome, bacterial diversity and biomarkers.

1 Supplementary Methods

1.1 Custom-made soil medium

Similar to previous studies on cultivation of microbial communities of soil (Liu *et al.*, 2013; Nguyen *et al.*, 2018), a custom-made soil medium (SM) was additionally formulated to mimic the nutritional conditions of the study sites. For SM, soil of a site highly similar to the experimental plots on the Swabian Alb was collected from the Botanical Garden of the University of Ulm, dried at room temperature and gently sieved through a ~2 mm sieve. 40 g of sieved soil were suspended in 200 ml distilled water and soluble components were dissolved by vigorous shaking for 30 min. The suspension was then centrifuged at 4200 rpm for 10 min. The pellet of solids was discarded and the supernatant (200 ml) was used to prepare 1 L of SM base solution. Finally, SM base solution was supplemented with 0.5 g L^{-1} of filter-sterilized glucose.

1.2 Cultivation of biomarker bacteria in microtiter plates

Growth of biomarker candidates in microtiter plates was tested in liquid media of R2A, NB and SM in microtiter plates using a BioTek Synergy H1M. Bacteria cells were prepared from 3 days culture in recommended media and cells were harvested by centrifugation at 4200 rpm for 10 min and resuspended in 1 ml of sterile distilled water (distilled water, autoclaved at 121 °C for 15 min). Cells were diluted to a final optical density at 600 nm (OD_{600}) of 0.1. 5 µL of the cell suspension was thoroughly mixed into 200 µL of media. All microplate-incubated cultures were conducted in 200 µL volumes in flat-bottomed polystyrene 96-well plates sealed with Breath-Easy® Sealing Membranes to facilitate gas exchange and incubated in the plate reader (BioTek Synergy H1M). Each treatment condition was plated in 96-well plates with 3 replicates. Wells containing media and no inoculants were used for media background correction. The plate was incubated at 30 ºC with continuous orbital shaking at 425 rpm using plate reader's internal temperature control and shaking functionality. OD_{600} was measured every four hours for 48 times points (2 days). The data was exported from microplate readers and OD_{600} values were background corrected by subtracting OD_{600} of sterile media.

2 Supplementary Tables

Table S1: NCBI accession number of the samples sequenced in the present study. All the samples are submitted under the BioProject accession PRJNA1188490.

Sample_ID	SRA accession	Bio Sample accession	library_layout	Instrument_model
C001	SRR31409978	SAMN44838193	PAIRED	Illumina MiSeq
C002	SRR31409967	SAMN44838194	PAIRED	Illumina MiSeq
C003	SRR31409956	SAMN44838195	PAIRED	Illumina MiSeq
C004	SRR31409849	SAMN44838196	PAIRED	Illumina MiSeq
C005	SRR31409838	SAMN44838197	PAIRED	Illumina MiSeq
C006	SRR31409827	SAMN44838198	PAIRED	Illumina MiSeq
C007	SRR31409944	SAMN44838199	PAIRED	Illumina MiSeq
C008	SRR31409933	SAMN44838200	PAIRED	Illumina MiSeq
C009	SRR31409921	SAMN44838201	PAIRED	Illumina MiSeq
C010	SRR31409910	SAMN44838202	PAIRED	Illumina MiSeq
C011	SRR31409899	SAMN44838203	PAIRED	Illumina MiSeq
C012	SRR31410222	SAMN44838204	PAIRED	Illumina MiSeq
C013	SRR31410211	SAMN44838205	PAIRED	Illumina MiSeq
C014	SRR31410200	SAMN44838206	PAIRED	Illumina MiSeq
C015	SRR31410189	SAMN44838207	PAIRED	Illumina MiSeq
C016	SRR31410178	SAMN44838208	PAIRED	Illumina MiSeq
C017	SRR31410167	SAMN44838209	PAIRED	Illumina MiSeq
C018	SRR31409822	SAMN44838210	PAIRED	Illumina MiSeq
C019	SRR31409810	SAMN44838211	PAIRED	Illumina MiSeq
C020	SRR31409799	SAMN44838212	PAIRED	Illumina MiSeq
C021	SRR31409884	SAMN44838213	PAIRED	Illumina MiSeq
C022	SRR31409873	SAMN44838214	PAIRED	Illumina MiSeq
C023	SRR31409862	SAMN44838215	PAIRED	Illumina MiSeq
C024	SRR31410153	SAMN44838216	PAIRED	Illumina MiSeq
C025	SRR31410142	SAMN44838217	PAIRED	Illumina MiSeq
C026	SRR31410131	SAMN44838218	PAIRED	Illumina MiSeq
C027	SRR31410024	SAMN44838219	PAIRED	Illumina MiSeq
C028	SRR31410013	SAMN44838220	PAIRED	Illumina MiSeq
C029	SRR31410007	SAMN44838221	PAIRED	Illumina MiSeq
C030	SRR31410006	SAMN44838222	PAIRED	Illumina MiSeq
C031	SRR31410005	SAMN44838223	PAIRED	Illumina MiSeq
C032	SRR31410004	SAMN44838224	PAIRED	Illumina MiSeq
C033	SRR31410003	SAMN44838225	PAIRED	Illumina MiSeq
C034	SRR31410002	SAMN44838226	PAIRED	Illumina MiSeq
C035	SRR31410001	SAMN44838227	PAIRED	Illumina MiSeq
C036	SRR31410000	SAMN44838228	PAIRED	Illumina MiSeq
C037	SRR31410127	SAMN44838229	PAIRED	Illumina MiSeq
C038	SRR31410126	SAMN44838230	PAIRED	Illumina MiSeq
C039	SRR31410124	SAMN44838231	PAIRED	Illumina MiSeq
C040	SRR31410123	SAMN44838232	PAIRED	Illumina MiSeq
C041	SRR31410122	SAMN44838233	PAIRED	Illumina MiSeq
C042	SRR31410121	SAMN44838234	PAIRED	Illumina MiSeq
C043	SRR31410120	SAMN44838235	PAIRED	Illumina MiSeq
C044	SRR31410119	SAMN44838236	PAIRED	Illumina MiSeq
C045	SRR31410118	SAMN44838237	PAIRED	Illumina MiSeq
C046	SRR31410117	SAMN44838238	PAIRED	Illumina MiSeq
C047	SRR31410116	SAMN44838239	PAIRED	Illumina MiSeq
C048	SRR31410115	SAMN44838240	PAIRED	Illumina MiSeq
C049	SRR31410113	SAMN44838241	PAIRED	Illumina MiSeq
C050	SRR31410112	SAMN44838242	PAIRED	Illumina MiSeq
C051	SRR31410111	SAMN44838243	PAIRED	Illumina MiSeq
C052	SRR31410110	SAMN44838244	PAIRED	Illumina MiSeq
C053	SRR31410109	SAMN44838245	PAIRED	Illumina MiSeq
C054	SRR31410108	SAMN44838246	PAIRED	Illumina MiSeq
C055	SRR31410107	SAMN44838247	PAIRED	Illumina MiSeq
C056	SRR31410106	SAMN44838248	PAIRED	Illumina MiSeq
C057	SRR31410105	SAMN44838249	PAIRED	Illumina MiSeq
C058	SRR31410104	SAMN44838250	PAIRED	Illumina MiSeq
C059	SRR31410102	SAMN44838251	PAIRED	Illumina MiSeq
C060	SRR31410101	SAMN44838252	PAIRED	Illumina MiSeq
C061	SRR31410100	SAMN44838253	PAIRED	Illumina MiSeq

C062	SRR31410099	SAMN44838254	PAIRED	Illumina MiSeq
C063	SRR31410098	SAMN44838255	PAIRED	Illumina MiSeq
C064	SRR31410097	SAMN44838256	PAIRED	Illumina MiSeq
C065	SRR31410096	SAMN44838257	PAIRED	Illumina MiSeq
C066	SRR31409999	SAMN44838258	PAIRED	Illumina MiSeq
C067	SRR31409998	SAMN44838259	PAIRED	Illumina MiSeq
C068	SRR31409997	SAMN44838260	PAIRED	Illumina MiSeq
C069	SRR31409995	SAMN44838261	PAIRED	Illumina MiSeq
C070	SRR31409994	SAMN44838262	PAIRED	Illumina MiSeq
C071	SRR31409993	SAMN44838263	PAIRED	Illumina MiSeq
C072	SRR31409992	SAMN44838264	PAIRED	Illumina MiSeq
C073	SRR31409991	SAMN44838265	PAIRED	Illumina MiSeq
C074	SRR31409990	SAMN44838266	PAIRED	Illumina MiSeq
C075	SRR31409989	SAMN44838267	PAIRED	Illumina MiSeq
C076	SRR31409988	SAMN44838268	PAIRED	Illumina MiSeq
C077	SRR31409987	SAMN44838269	PAIRED	Illumina MiSeq
C078	SRR31409986	SAMN44838270	PAIRED	Illumina MiSeq
C079	SRR31410284	SAMN44838271	PAIRED	Illumina MiSeq
C080	SRR31410283	SAMN44838272	PAIRED	Illumina MiSeq
C081	SRR31410282	SAMN44838273	PAIRED	Illumina MiSeq
C082	SRR31410281	SAMN44838274	PAIRED	Illumina MiSeq
C083	SRR31410280	SAMN44838275	PAIRED	Illumina MiSeq
C084	SRR31410279	SAMN44838276	PAIRED	Illumina MiSeq
C085	SRR31410278	SAMN44838277	PAIRED	Illumina MiSeq
C086	SRR31410277	SAMN44838278	PAIRED	Illumina MiSeq
C087	SRR31410276	SAMN44838279	PAIRED	Illumina MiSeq
C088	SRR31410275	SAMN44838280	PAIRED	Illumina MiSeq
C089	SRR31410273	SAMN44838281	PAIRED	Illumina MiSeq
C090	SRR31410272	SAMN44838282	PAIRED	Illumina MiSeq
C091	SRR31410271	SAMN44838283	PAIRED	Illumina MiSeq
C092	SRR31410270	SAMN44838284	PAIRED	Illumina MiSeq

Table S2: Results of generalized linear mixed models (GLMMs) testing the effect of organic fertilizer on Pielou's evenness. Estimates (±SE), test statistic, p-value, and 95% confidence intervals (CI) are shown. Reference level = CS (no fertilization).

Response variable	Treatment	Estimate	SE	Statistic	p-value	95% CI (low–high)
Pielou's evenness	Intercept (CS)	-0.1121	0.013	-8.34	<0.001	-0.138 – -0.086
	BD	-0.0153	0.019	-0.81	0.420	-0.053 – 0.022
	CM	-0.0169	0.02	-0.85	0.398	-0.056 – 0.022
	PS	0.0165	0.019	0.87	0.386	-0.021 – 0.054

Table S3: Results of generalized linear mixed models (GLMMs) testing the effect of organic fertilizer key metabolic pathways. Estimates are reported with standard error (SE), test statistic, p-values, and 95% confidence intervals (CI). Reference level = control (no fertilization).

Response variable	Treatment	Estimate	SE	Statistic	p-value	95% CI (low–high)
Carbohydrate Metabolism	Intercept (CS)	-0.412	0.064	-6.431	0.000	-0.538- -0.287
	BD	-0.311	0.091	-3.433	0.001	-0.489- -0.134
	CM	-0.230	0.095	-2.414	0.016	-0.416- -0.043
	PS	-0.317	0.091	-3.503	0.000	-0.495- -0.140
Methane Metabolism	Intercept (CS)	0.237	0.057	4.124	0.000	0.124- 0.350
	BD	-0.158	0.081	-1.944	0.052	-0.317- 0.001
	CM	-0.060	0.085	-0.707	0.480	-0.227- 0.107
	PS	-0.081	0.081	-0.991	0.322	-0.240- 0.079
Nitrogen Metabolism	Intercept (CS)	-0.361	0.060	-6.020	0.000	-0.479- -0.244
	BD	-0.218	0.085	-2.572	0.010	-0.385- -0.052
	CM	-0.134	0.089	-1.509	0.131	-0.309- 0.040
	PS	-0.230	0.085	-2.710	0.007	-0.396- -0.064
Sulfur Metabolism	Intercept (CS)	-1.567	0.058	-27.139	0.000	-1.680- -1.453
	BD	-0.195	0.082	-2.388	0.017	-0.355- -0.035
	CM	-0.122	0.086	-1.423	0.155	-0.290- 0.046
	PS	-0.209	0.082	-2.561	0.010	-0.369- -0.049

Table S4: Results of GLMMs testing the effect of fertilizer treatments on biomarker bacteria. Estimates (±SE), test statistic, p-value, and 95% confidence intervals (CI) are shown. Reference level = CS (no fertilization).

Response variable	Treatment	Estimate	SE	Statistic	p-value	95% CI (low–high)
Bacillus	Intercept (CS)	-2.829	0.178	-15.892	0.000	-3.178- -2.480
	BD	-0.761	0.252	-3.015	0.003	-1.256- -0.266
	CM	-1.007	0.264	-3.814	0.000	-1.524- -0.489
	PS	-1.380	0.253	-5.465	0.000	-1.876- -0.885
Bradyrhizobium	Intercept (CS)	-3.761	0.081	-46.159	0.000	-3.921- -3.601
	BD	-0.339	0.115	-2.940	0.003	-0.565- -0.113
	CM	-0.156	0.121	-1.289	0.197	-0.393- 0.081
	PS	-0.222	0.115	-1.923	0.054	-0.447- 0.004
Gaiella	Intercept (CS)	-3.809	0.079	-48.436	0.000	-3.964- -3.655
	BD	0.396	0.108	3.662	0.000	0.184- 0.608
	CM	0.386	0.113	3.411	0.001	0.164- 0.608
	PS	0.671	0.107	6.295	0.000	0.462- 0.880
Nocardioides	Intercept (CS)	-4.652	0.077	-60.246	0.000	-4.803- -4.501
	BD	0.053	0.109	0.490	0.624	-0.161- 0.268
	CM	0.025	0.115	0.222	0.825	-0.199- 0.250
	PS	0.315	0.109	2.886	0.004	0.101- 0.529
Pseudonocardia	Intercept (CS)	-4.253	0.082	-51.635	0.000	-4.414- -4.091
	BD	-0.016	0.116	-0.136	0.892	-0.244- 0.212
	CM	0.123	0.122	1.013	0.311	-0.115- 0.361
	PS	0.526	0.115	4.568	0.000	0.300- 0.751
Reyranella	Intercept (CS)	-4.332	0.041	-104.46	0.000	-4.414- -4.251
	BD	-0.253	0.058	-4.341	0.000	-0.367- -0.139
	CM	-0.203	0.061	-3.325	0.001	-0.323- -0.083
	PS	-0.138	0.058	-2.373	0.018	-0.252- -0.024
Solirubrobacter	Intercept (CS)	-4.588	0.061	-75.641	0.000	-4.707- -4.469
	BD	0.018	0.085	0.210	0.834	-0.149- 0.184
	CM	-0.029	0.089	-0.325	0.745	-0.204- 0.146
	PS	0.230	0.084	2.735	0.006	0.065- 0.394

Table S5: Selected type strains, media recommended by the supplier and growth on agar plates of six commonly used complex media for cultivation of a wide range of bacteria. Growth or no growth are indicated by "+" and "-", respectively; n.d.: not determined.

Type strain	BHI	TSA	LB	2xTY	NA	R2A	SM
Gaiella occulta CECT 7815 ^{T}	n.d.	n.d.	n.d.	n.d.	n.d.	n.d.	n.d.
Reyranella soli NBRC 108950^{T}	n.d.	n.d.	n.d.	n.d.	n.d.	n.d.	n.d.
Bacillus subtilis DSM 10T	+	+	+	+	+	+	+
Bradyrhizobium japonicum DSM 30131^{T}	-	-	-	-	+	+	+
Nocardioides albus DSM 43109^{T}	+	+	+	+	+	+	+
Pseudonocardia antarctica DSM 44749^{T}	+	+	+	+	+	+	+
Solirubrobacter pauli DSM 14954^{T}	-	-	-	-	+	+	+
strains with growth on medium	3	3	3	3	5	5	5

Table S6: Results of linear mixed-effects model (LME) testing the effect of fertilizer treatments, mean abundance of biomarker bacteria, and functional diversity. Estimates (±SE), test statistic, p-value, and 95% confidence intervals (CI) are reported. Reference level = CS (no fertilization).

Response variable	Treatment	Estimate	SE	Statistic	p-value	95% CI (low–high)
Bacillus	Intercept (CS)	7.796	0.010	781.037	0.000	7.776- 7.816
	mean_abund	0.000	0.000	-1.344	0.184	0.000- 0.000
	BD	-0.032	0.013	-2.524	0.021	-0.059- -0.005
	CM	-0.047	0.013	-3.728	0.001	-0.074- -0.021
	PS	-0.074	0.012	-6.069	0.000	-0.100- -0.049
	mean_abund:BD	0.000	0.000	0.489	0.626	0.000- 0.000
	mean_abund:CM	0.000	0.000	2.404	0.019	0.000- 0.000
	mean_abund:PS	0.001	0.000	3.931	0.000	0.000- 0.001
Bradyrhizobium	Intercept (CS)	7.786	0.016	490.597	0.000	7.754- 7.818
	mean_abund	0.000	0.000	-0.069	0.945	0.000- 0.000
	BD	-0.045	0.021	-2.108	0.049	-0.090- 0.000
	CM	-0.037	0.022	-1.726	0.101	-0.083- 0.008
	PS	-0.068	0.021	-3.249	0.004	-0.112- -0.024
	mean_abund:BD	0.000	0.000	1.367	0.176	0.000- 0.000
	mean_abund:CM	0.000	0.000	0.632	0.530	0.000- 0.000
	mean_abund:PS	0.000	0.000	1.360	0.178	0.000- 0.000
Solirubrobacter	Intercept (CS)	7.783	0.011	681.531	0.000	7.760- 7.805
	mean_abund	0.000	0.000	0.245	0.807	-0.001- 0.001
	BD	-0.036	0.016	-2.200	0.040	-0.069- -0.002
	CM	-0.024	0.016	-1.470	0.158	-0.059- 0.010
	PS	-0.018	0.016	-1.090	0.289	-0.052- 0.016
	mean_abund:BD	0.000	0.001	0.854	0.396	-0.001- 0.001
	mean_abund:CM	0.000	0.001	-0.117	0.907	-0.001- 0.001
	mean_abund:PS	-0.001	0.000	-1.539	0.129	-0.002- 0.000
Nocardioides	Intercept (CS)	7.786	0.011	717.211	0.000	7.764- 7.807
	mean_abund	0.000	0.001	-0.051	0.960	-0.001- 0.001
	BD	-0.035	0.016	-2.237	0.037	-0.067- -0.002
	CM	-0.018	0.017	-1.039	0.312	-0.053- 0.018
	PS	-0.037	0.015	-2.536	0.020	-0.068- -0.007
	mean_abund:BD	0.001	0.001	0.854	0.396	-0.001- 0.002
	mean_abund:CM	0.000	0.001	-0.554	0.582	-0.002- 0.001
	mean_abund:PS	0.000	0.001	-0.507	0.614	-0.002- 0.001

3 Supplementary Figures

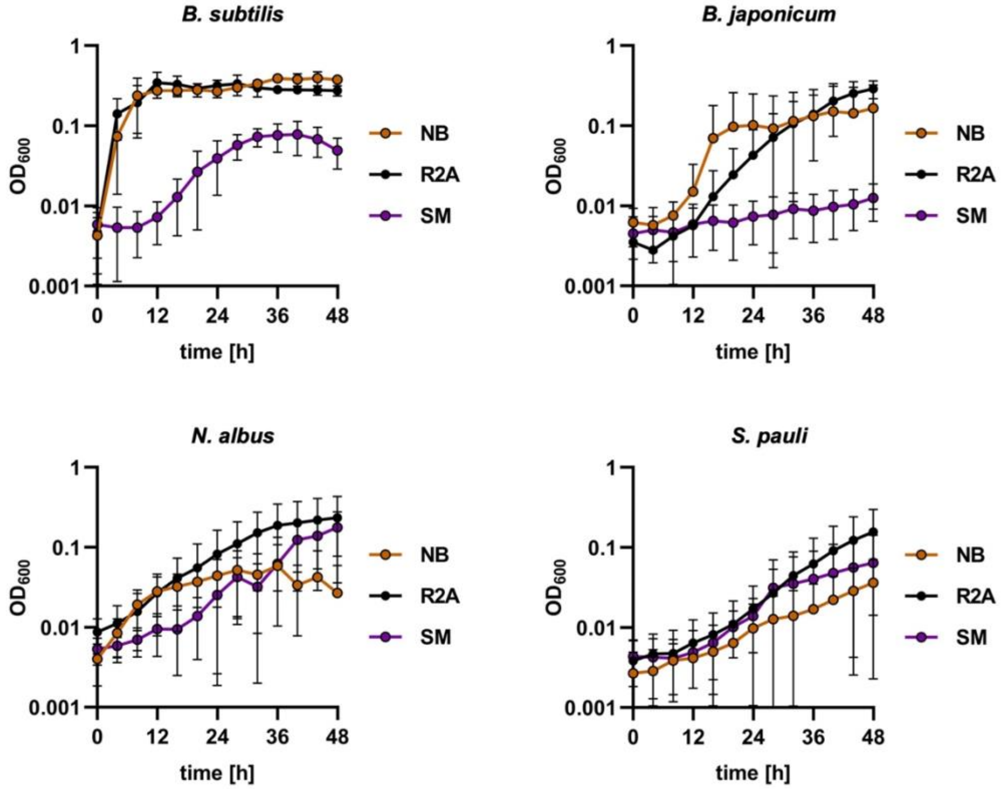

Figure S1: Growth (OD_{600}) of *B. subtilis* DSM 10, *B. japonicum* DSM 30131, *N. albus* DSM 43109, and *S. pauli* DSM 14954 on nutrient broth (NB; orange), R2A (black), or soil medium (SM; purple) in microtiter plates at 30 °C with agitation. All values are mean ± standard deviation of n = 8-9 independent cultures per strain.

4 Supplementary References

Liu, X., Song, Q., Tang, Y., Li, W., Xu, J., Wu, J., et al. (2013) Human health risk assessment of heavy metals in soil-vegetable system: a multi-medium analysis. *Sci Total Environ* **463–464**: 530–540.

Nguyen, T.M., Seo, C., Ji, M., Paik, M.-J., Myung, S.-W., and Kim, J. (2018) Effective Soil Extraction Method for Cultivating Previously Uncultured Soil Bacteria. *Appl Environ Microbiol* **84**: e01145-18.
